# Consistency of effect of ezetimibe/simvastatin compared with intensified lipid-lowering treatment strategies in obese and non-obese diabetic subjects

**DOI:** 10.1186/1476-511X-12-103

**Published:** 2013-07-16

**Authors:** Jeffrey B Rosen, Jose G Jimenez, Valdis Pirags, Hella Vides, Rachid Massaad, Mary E Hanson, Philippe Brudi, Joseph Triscari

**Affiliations:** 1 Clinical Research of South Florida, Coral Gables, FL, USA; 2 Hospital CIMA San Jose, Escazu, San Jose, Costa Rica; 3 University of Latvia, Riga, Latvia; 4 Viljandi Hospital, Vildjandimaa, Estonia; 5 MSD Belgium, Brussels, Belgium; 6 Merck & Co, Inc., Whitehouse Station, NJ, USA

**Keywords:** Atorvastatin, Ezetimibe, Diabetes, Obesity, Rosuvastatin, Statin

## Abstract

**Purpose:**

This *post hoc* analysis assessed switching to ezetimibe/simvastatin 10/20 mg vs doubling the baseline statin dose to simvastatin 40 mg or atorvastatin 20 mg or switching to rosuvastatin 10 mg in subgroups of obese (BMI ≥30 kg/m^2^) and non-obese (BMI <30 kg/m^2^) diabetic subjects.

**Methods:**

This was a randomized, double-blind, 12-week study of adults 18–79 years with cardiovascular disease with low-density lipoprotein cholesterol (LDL-C) ≥70 and ≤160 mg/dl. Percent change in LDL-C and other lipids was estimated.

**Results:**

In obese subjects (n = 466), percent changes in LDL-C and most other lipids were greater with ezetimibe/simvastatin vs doubling the baseline statin dose or switching to rosuvastatin. In non-obese subjects (n = 342), percent changes in LDL-C, total cholesterol, non-HDL-C, Apo B and Apo A-I were greater with ezetimibe/simvastatin vs doubling the baseline statin dose or switching to rosuvastatin; and treatment with ezetimibe/simvastatin resulted in greater changes in triglycerides vs rosuvastatin and HDL-C vs doubling the baseline statin dose. The safety profiles were generally similar.

**Conclusions:**

Regardless of baseline obesity status, switching to ezetimibe/simvastatin was more effective at reducing LDL-C, total cholesterol, non-HDL-C, and Apo B vs doubling the baseline statin dose to simvastatin 40 mg or atorvastatin 20 mg or switching to rosuvastatin 10 mg.

## Background

The presence of diabetes and obesity is associated with an increased risk of cardiovascular disease (CVD) [1-3]. Recommendations from the US, Canadian, and European treatment guidelines focus on reducing LDL-C to <70 mg/dL (<1.81 mmol/L) for high risk patients with diabetes and cardiovascular disease (CVD) [4-6]. However, in obese individuals, when dyslipidemia is present it is often characterized by decreased HDL-C and increased non-HDL-C, triglycerides, apolipoprotein (Apo) B and small, dense LDL-C particles, although often, relatively normal levels of LDL-C [7,8]. Likewise, the dyslipidemia associated with diabetes is characterized by a similar lipid profile [9]. As a result, individuals with diabetes and/or obesity with dyslipidemia may be undertreated even after intense lipid-lowering therapy [9]. The use of combination therapies may be warranted to achieve optimal lipid and lipoprotein levels, as well as treatment targets for Apo B and non-HDL-C, as recommended by the American Diabetes Association and American College of Cardiology consensus statement for patients with elevated cardiometabolic risk [9].

Clinical trials done in diabetic subjects have shown that the combination of ezetimibe/simvastatin provides additional lipid reductions over simvastatin and atorvastatin monotherapy [10,11]. It has also been shown that a higher proportion of high risk CVD patients, including those with diabetes, achieve target LDL-C levels when treated with the combination of ezetimibe/simvastatin 10/40 mg compared with switching to atorvastatin 40 mg or rosuvastatin 5–10 mg [12]. In addition, a pooled analysis of 27 studies that grouped subjects by baseline diabetic status (with or without diabetes) showed that the lipid profile in subjects with diabetes improved to a greater extent than in subjects without diabetes after treatment with the combination of ezetimibe/statin [13]. However, data assessing the effect of lipid lowering therapies in obese and non-obese diabetic patients are limited.

The primary objective of this *post hoc* analysis was to assess the consistency of treatment effect of switching to ezetimibe/simvastatin 10/20 mg vs. doubling the baseline statin dose to simvastatin 40 mg or atorvastatin 20 mg in subgroups of obese diabetic subjects (n = 466) and non-obese diabetic subjects (n = 342) based on body mass index (BMI) ≥30 kg/m^2^ or <30 kg/m^2^. The secondary objective was to perform a similar *post hoc* analysis as the primary for the comparison of ezetimibe/simvastatin 10/20 mg vs switching to rosuvastatin 10 mg in the same subgroups of subjects. Tolerability was also assessed.

## Methods

This was a *post hoc* analysis of a randomized, double-blind, 12-week study in subgroups of obese and non-obese diabetic subjects based on body mass index ≥30 kg/m^2^ or <30 kg/m^2^ (Protocol 133; clinical trials registry NCT00862251) [14]. The study was carried out between June 2009 and March 2011 in 86 centers in Austria, Bulgaria, Chile, Costa Rica, Croatia, Egypt, Estonia, Germany, Greece, Hungary, Italy, Latvia, Lithuania, Peru, Portugal, and the United States and was conducted in conformance with Good Clinical Practice standards and applicable country and/or local statutes and regulations regarding ethical committee review, informed consent, and the protection of human subjects participating in biomedical research.

### Subjects

Eligible subjects were non-Asian males or females, ≥18 and <80 years, with type 1 or type 2 diabetes mellitus (HbA1c ≤8.5%) and symptomatic/overt CVD who were naïve to statin and/or ezetimibe or were taking a stable dose of approved lipid-lowering therapy (simvastatin 10 or 20 mg; atorvastatin 10 mg; pravastatin 10, 20 or 40 mg; fluvastatin 20, 40 or 80 mg, ezetimibe 10 mg; lovastatin 10, 20, 40 or 80 mg, or ezetimibe + fluvastatin 10 or 20 mg) and if needed, taking a stable anti-diabetic medication for 3 months prior to the screening visit. Subjects must have been willing to maintain a cholesterol- and glucose-lowering diet for the duration of the study. Prior to randomization subjects were required to complete the screening/stabilization period on simvastatin 20 mg or atorvastatin 10 mg with LDL-C ≥70 mg/dl (1.81 mmol/L) and ≤160 mg/dl (4.14 mmol/L), alanine transaminase (ALT) and aspartate aminotransferase (AST) ≤2.0 x upper limit of normal (ULN) (no active liver disease), creatine kinase (CK) ≤3 x ULN, and triglycerides ≤400 mg/dl (4.52 mmol/L). Subjects were excluded if they were Asian, since rosuvastatin prescribing information recommends a 5 mg starting dose for Asians. Subjects were also excluded if they had uncontrolled endocrine or metabolic disease that impacted lipids/lipoproteins, uncontrolled or recent-onset diabetes, congestive heart failure, hypertension, digestive disease/intestinal malabsorption, were taking agents impacting lipids, potent CYP3A4 inhibitors, >1 quart/day grapefruit juice, systemic corticosteroids, cyclosporine, danazol or fusidic acid, agents increasing risk of myopathy, or warfarin.

### Randomization and blinding

After a 6-week run-in period of simvastatin 20 mg or atorvastatin 10 mg (baseline statin doses), subjects with LDL-C ≥70 mg/dL (1.81 mmol/L) and ≤160 mg/dL (4.14 mmol/L) were stratified according to their baseline statin and randomized in a 2:1:2 ratio within strata to ezetimibe/simvastatin 10/20 mg, doubling their baseline statin, or rosuvastatin 10 mg for 6 weeks using an interactive voice response system. Subjects who met eligibility criteria at the screening visit were provided with open-label simvastatin 20 mg or atorvastatin 10 mg tablets. At randomization, subjects were supplied in a double dummy fashion with bottles of blinded ezetimibe/simvastatin 10/20 mg or matching placebo and rosuvastatin 10 mg or matching placebo. A blocked randomization was used with a block size of 5. Subjects, investigators, and study personnel involved in the study remained blinded during the study period until the data were complete and clean and a database lock was obtained.

### Efficacy endpoints

In this *post hoc* analysis, the primary evaluation was the consistency of the treatment effect between ezetimibe/simvastatin 10/20 mg vs. doubling the baseline statin dose across subgroups (obese/non-obese) and the secondary evaluation was the consistency of the treatment effect between ezetimibe/simvastatin 10/20 mg vs. rosuvastatin 10 mg across subgroups (obese/non-obese). Efficacy endpoints of interest were the percent change from baseline in low density lipoprotein cholesterol (LDL-C), total cholesterol, triglycerides, high-density lipoprotein cholesterol (HDL-C), non-HDL-C, Apo B, Apo A-I, high-sensitivity C-reactive protein (hs-CRP), LDL-C/HDL-C ratio, total cholesterol/HDL-C ratio, non-HDL-C/HDL-C ratio, and Apo B/Apo A-I ratio at Week 6. The percent of patients achieving LDL-C <70 mg/dl (1.81 mmol/l), non-HDL-C <100 mg/dl (2.59 mmol/l), or Apo B <80 mg/dl (0.80 g/L) was assessed at Week 6.

### Safety endpoints

Prespecified adverse events (AEs) of interest were gastrointestinal-related, gallbladder-related, allergic reaction/rash-related, and hepatitis-related AEs; consecutive elevations in alanine aminotransferase (ALT) / aspartate aminotransferase (AST) ≥3 x ULN, ≥5 x ULN, and ≥10 x ULN; consecutive elevations in ALT and/or AST ≥3 x ULN, ≥5 x ULN and ≥10 x ULN, elevations in ALT or AST ≥3 x ULN, elevations in CK ≥10 x ULN, elevations in CK ≥10 x ULN with muscle symptoms, and elevations in CK ≥10 x ULN with muscle symptoms that are considered drug-related. In addition, the broad AE categories consisting of the percentage of patients with any AE, a drug-related AE, a serious AE, a serious drug-related AE, and who discontinued due to an AE were assessed.

### Statistics

The full analysis set (FAS), which included all randomized patients who took at least 1 dose of study drug and had a baseline measurement, was used for the efficacy analyses. The all-patients-as-treated (APaT) approach was used for the safety analyses, and included all randomized patients receiving ≥1 dose of study drug and all safety data up to 14 days after the last intake of study medication. At least 1 laboratory/vital sign measurement was required subsequent to at least 1 dose of study treatment for inclusion in the analysis of each specific parameter. The estimate of the within-group treatment effect, and the between-group treatment effect with a nominal 95% confidence interval for the efficacy variables was estimated within each subgroup (i.e., obese/non-obese) using a constrained longitudinal data analysis model applied to each subgroup separately, with terms for treatment, time, time-by-treatment interaction, stratum, and time-by-stratum interaction. As some deviation from normality was observed for the percent change from baseline in LDL-C, a similar *post hoc* sensitivity analysis on log-transformed data, as done for the overall population, was performed to corroborate the main analysis on un-transformed data [14]. For AEs of interest and broad AE categories, count and % of patients with AEs were provided by treatment group within each subgroup.

## Results

**Figure 1:**
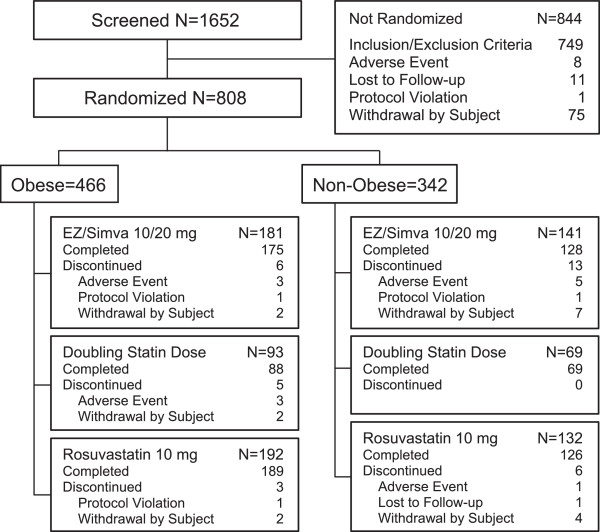
Flow of subjects through the study.

The flow of subjects through the study is shown in Figure 1. Of the 808 subjects that were randomized, 466 (57.7%) were included in the obese subgroup and 342 (42.3%) were included in the non-obese subgroup. Within the obese subgroup, 181 (38.8%) were randomized to ezetimibe/simvastatin, 93 (20.0%) were randomized to have their statin dose doubled and 192 (41.2%) were randomized to rosuvastatin 10 mg. Within the non-obese subgroup, 141 (41.2%) were randomized to ezetimibe/simvastatin, 69 (20.2%) were randomized to have their statin dose doubled and 132 (38.6%) were randomized to rosuvastatin 10 mg.

Baseline demographics and clinical characteristics were generally similar between obese and non-obese subjects with a few exceptions (Table 1). In obese subjects, the mean age (±SD) was ~63 (±8) years and the majority of subjects were Caucasian in all 3 treatment groups. In the subgroup of obese subjects, the rosuvastatin treatment group had a slightly higher proportion of males (53.1%) compared with the ezetimibe/simvastatin 10/20 mg (49.7%) and doubling the statin dose (49.5%) treatment groups. In non-obese subjects, the mean age (±SD) ranged from 64 (±9) years to 66 (±8) years and the majority of subjects were Caucasian in all 3 treatment groups. There was a higher proportion of Hispanics or Latinos in the non-obese subgroup (23.2% in the doubling statin group, 24.2% in the rosuvastatin 10 mg group and 31.9% in the ezetimibe/simvastatin 10/20 mg group) compared with the obese subgroup (9.7% in the doubling statin group, 10.5% in the ezetimibe/simvastatin 10/20 mg group and 11.5% in the rosuvastatin group). Triglyceride levels were higher in obese subjects in all 3 treatment groups (median = 149 mg/dL, 136 mg/dL and 143.5 mg/dL in the ezetimibe/simvastatin 10/20 mg, doubling the statin dose and rosuvastatin 10 mg groups, respectively) compared with non-obese subjects (median = 126 mg/dL, 126 mg/dL and 132 mg/dL in the ezetimibe/simvastatin 10/20 mg, doubling the statin dose and rosuvastatin 10 mg groups, respectively). Finally, hs-CRP levels were higher in obese subjects compared with non-obese subjects taking ezetimibe/simvastatin 10/20 mg (2.4 mg/L vs 1.6 mg/L) and rosuvastatin 10 mg (2.6 mg/L vs 1.6 mg/L) but not in subjects whose baseline statin dose was doubled (1.9 mg/L vs 2.0 mg/L).

**Table 1:** Baseline demographics and clinical characteristics

	**EZ/Simva 10/20 mg**		**Doubling statin dose**		**Rosuvastatin 10 mg**	
**Obese subjects**	**n = 181**		**n = 93**		**n = 192**	
**Sex, n (%)**						
Male	90	(49.7)	46	(49.5)	102	(53.1)
Female	91	(50.3)	47	(50.5)	90	(46.9)
**Age, years Mean (SD)**	63.3	(8.2)	63.5	(8.5)	63.3	(8.2)
**Weight, kg Mean (SD)**	99.3	(15.3)	96.2	(12.5)	96.5	(13.3)
**Body Mass Index (BMI, kg/m**^**2**^**)**						
Mean (SD)	35.4	(4.3)	34.6	(3.7)	34.6	(3.7)
**Race, n (%)**						
American Indian or Alaska Native	1	(0.6)	0	(0.0)	1	(0.5)
Black or African American	4	(2.2)	1	(1.1)	1	(0.5)
Multi-Racial	10	(5.5)	4	(4.3)	8	(4.2)
White	166	(91.7)	88	(94.6)	182	(94.8)
**Ethnicity, n (%)**						
Hispanic or Latino	19	(10.5)	9	(9.7)	22	(11.5)
Not Hispanic or Latino	162	(89.5)	84	(90.3)	170	(88.5)
**Clinical Characteristics, mg/dL**	**Mean**	**SD**	**Mean**	**SD**	**Mean**	**SD**
LDL-C	97.9	22.4	99.3	22.0	95.7	19.1
Total cholesterol	179.0	30.4	180.5	27.0	178.3	24.2
Triglycerides*	149.0	74.4	136.0	77.2	143.5	74.4
HDL-C	48.6	11.5	51.0	11.6	47.8	10.7
Non-HDL-C	130.4	28.3	129.4	25.8	127.5	23.6
Apo B	101.6	19.8	101.7	17.7	100.0	18.6
Apo A-I	148.4	24.6	153.5	22.2	147.6	22.0
hs-CRP*, mg/L	2.4	2.9	1.9	3.2	2.6	3.7
**Non-obese subjects**	**n = 141**		**n = 69**		**n = 132**	
**Sex, n (%)**						
Male	70	(49.6)	34	(49.3)	80	(60.6)
Female	71	(50.4)	35	(50.7)	52	(39.4)
**Age, years** Mean (SD)	65.2	(9.3)	66.2	(7.9)	64.0	(8.6)
**Weight, kg** Mean (SD)	73.5	(11.0)	76.3	(11.5)	76.3	(11.5)
**Body mass index (BMI, kg/m**^**2**^**)**						
Mean (SD)	35.4	(4.3)	34.6	(3.7)	34.6	(3.7)
**Race, n (%)**						
American Indian or Alaska Native	3	(2.1)	2	(2.9)	4	(3.0)
Black or African American	1	(0.7)	1	(1.4)	1	(0.8)
Multi-Racial	23	(16.3)	7	(10.1)	16	(12.1)
White	114	(80.9)	59	(85.5)	111	(84.1)
**Ethnicity, n (%)**						
Hispanic or Latino	45	(31.9)	16	(23.2)	32	(24.2)
Not Hispanic or Latino	96	(68.1)	53	(76.8)	100	(75.8)
**Clinical characteristics, mg/dL**	**Mean**	**SD**	**Mean**	**SD**	**Mean**	**SD**
LDL-C	100.1	21.6	94.7	20.2	99.9	20.5
Total cholesterol	180.5	30.9	173.0	26.3	178.7	28.7
Triglycerides*	126.0	65.1	126.0	51.2	132.0	76.3
HDL-C	52.6	15.8	50.6	12.7	50.5	13.3
Non-HDL-C	127.9	26.9	122.4	25.3	128.2	26.4
Apo B	101.7	20.4	98.9	20.1	101.0	20.0
Apo A-I	152.7	32.9	153.1	28.2	149.4	27.3
hs-CRP*, mg/L	1.6	2.9	2.0	2.2	1.6	2.3

*Data for TG and hs-CRP are presented as median and robust standard deviation.

*EZ* Ezetimibe, *Simva* Simvastatin.

**Figure 2:**
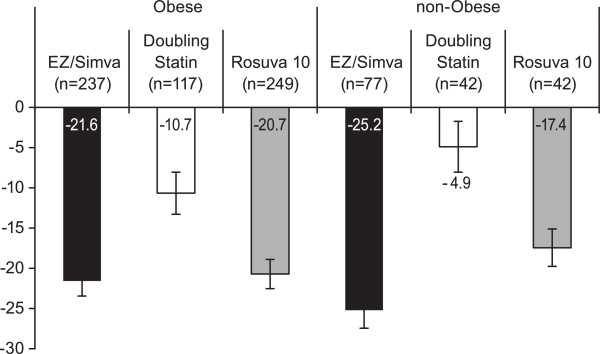
**Least squares mean percent change from baseline in LDL-C in obese and non-obese subjects after 6 weeks of treatment.** Bars represent standard error. (FAS population).

Regardless of baseline obesity status (obese/non-obese), in this population of high risk diabetic subjects, switching to ezetimibe/simvastatin 10/20 mg generally resulted in numerically greater changes in LDL-C compared with doubling the baseline statin dose or switching to rosuvastatin, though the treatment effect was smaller for the comparison of ezetimibe/simvastatin 10/20 mg vs. rosuvastatin 10 mg in the obese subgroup (Figure 2). In obese subjects LS mean percent changes from baseline in LDL-C were −21.6%, -10.7%, and −20.7% in the ezetimibe/simvastatin 10/20 mg group, in the doubling statin group, and in the rosuvastatin 10 mg group, respectively. In non-obese subjects, LS mean percent changes from baseline in LDL-C were −25.2%, -4.9%, and −17.4% in the ezetimibe/simvastatin 10/20 mg group, in the doubling statin group, and in the rosuvastatin 10 mg group, respectively. The results of the *post hoc* sensitivity analysis on log-transformed data were consistent with those of the main analysis on un-transformed data; however, for the comparison vs. rosuvastatin in obese subjects, the magnitude of the LDL-C difference in the post hoc sensitivity analysis was somewhat higher compared with the main analysis (−3.2% in this analysis vs. -0.9% in the main analysis).

**Figure 3:**
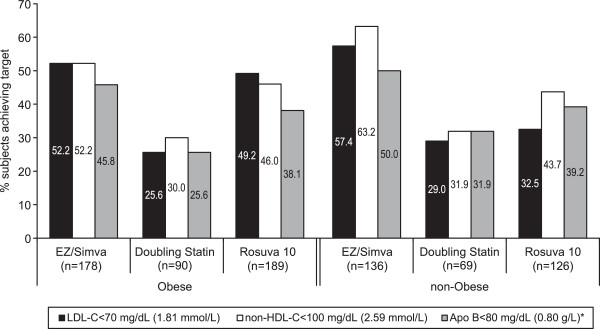
Percent of subjects achieving specified targets after 6 weeks of treatment (FAS population).

Achievement of specified lipid targets is shown in Figure 3. In obese and non-obese subjects, more subjects achieved the specified LDL-C targets of <70 mg/dL when treated with ezetimibe/simvastatin 10/20 mg (non-obese: 57.4%; obese: 52.2%) compared with doubling the baseline statin dose to simvastatin 40 mg or atorvastatin 20 mg (non-obese: 29.0%; obese: 25.6%) or switching to rosuvastatin 10 mg (non-obese: 32.5%; obese: 49.2%). Similarly, more subjects achieved non-HDL-C <100 mg/dL when treated with ezetimibe/simvastatin 10/20 mg (non-obese: 63.2%; obese: 52.2%) compared with doubling the baseline statin dose to simvastatin 40 mg or atorvastatin 20 mg (non-obese: 31.9%; obese: 30.0%) or switching to rosuvastatin 10 mg (non-obese: 43.7%; obese: 46.0%; Figure 3). Finally, a greater percentage of subjects achieved Apo B <80 mg/dL when treated with ezetimibe/simvastatin 10/20 mg (non-obese: 50.0%; obese: 45.8%) compared with doubling the baseline statin dose to simvastatin 40 mg or atorvastatin 20 mg (non-obese: 31.9%; obese: 25.6%) or switching to rosuvastatin 10 mg (non-obese: 39.2%; obese: 38.1%; Figure 3).

**Figure 4:**
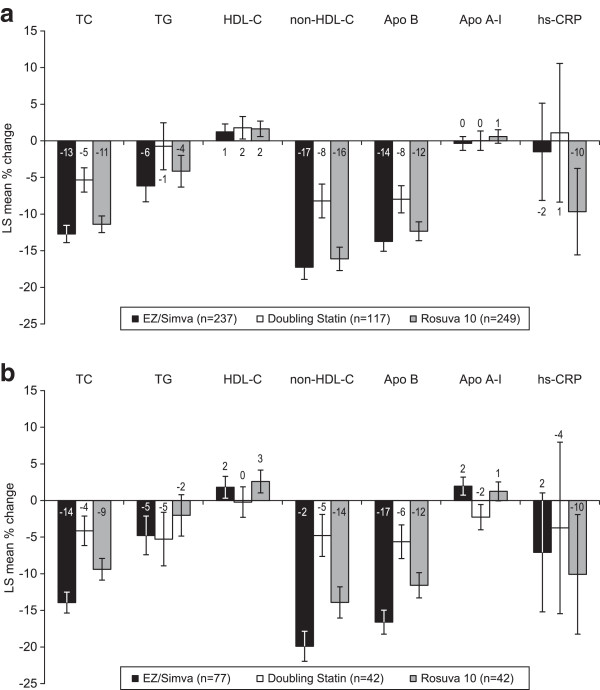
**Least squares mean percent change from baseline in lipids, lipoproteins and hs-CRP after 6 weeks of treatment.** Bars represent standard error. (FAS population). **a**. Obese subjects. **b**. non-obese subjects.

**Figure 5:**
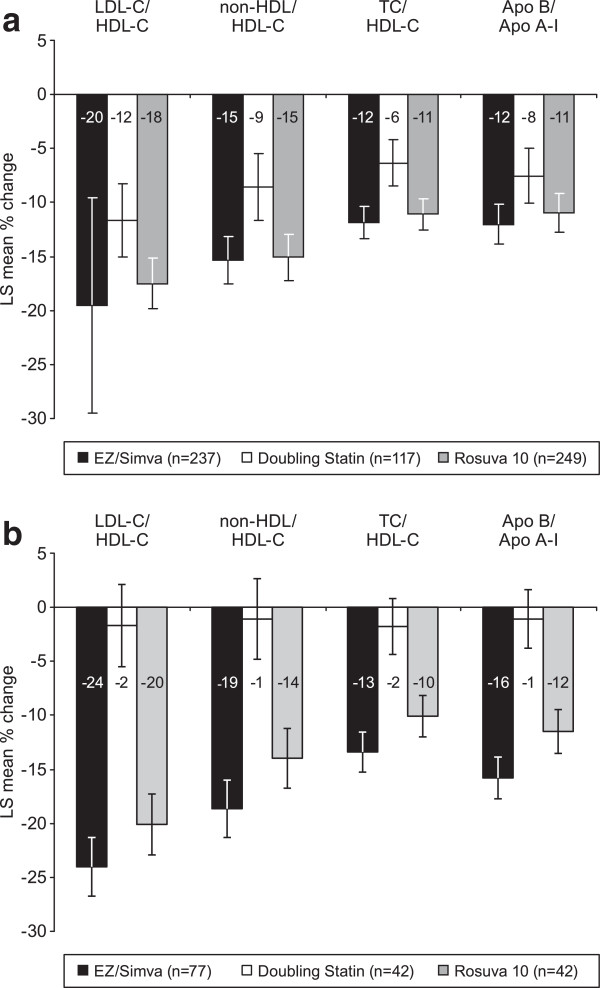
**Least squares mean percent change from baseline in lipid ratios after 6 weeks of treatment.** Bars represent standard error. (FAS population). **a**. obese subjects. **b**. non-obese subjects.

In both obese and non-obese subjects, treatment with ezetimibe/simvastatin 10/20 mg resulted in numerically greater changes in total cholesterol, non-HDL-C, and Apo B compared with doubling the baseline statin dose to simvastatin 40 mg or atorvastatin 20 mg or vs switching to rosuvastatin 10 mg (Table 2 and Figures 4a and 4b). However, changes in HDL-C and Apo A-I appeared to be similar between treatments in obese subjects (Figure 4a). In non-obese subjects (Figure 4b) changes in triglycerides were numerically greater in the ezetimibe/simvastatin 10/20 treatment group compared with the rosuvastatin 10 mg group, while the subjects whose baseline statin dose was doubled showed similar decreases to subjects treated with ezetimibe/simvastatin 10/20 mg. In non-obese subjects, changes in HDL-C were similar between treatment groups, and increases in Apo A-I were greater in the ezetimibe/simvastatin 10/20 mg vs the doubling the baseline statin dose group (Figure 4b). In both obese and non-obese subjects changes in hs-CRP were numerically greater with rosuvastatin 10 mg vs ezetimibe/simvastatin 10/20 mg (Figures 4a and 4b). In both obese and non-obese subjects, ezetimibe/simvastatin 10/20 mg was more effective at improving lipid ratios compared with doubling the baseline statin dose to simvastatin 40 mg or atorvastatin 20 mg, although the changes were similar to those of rosuvastatin 10 mg-treated subjects in both obese and non-obese subjects (Figures 5a and 5b).

**Table 2:** Least Squares mean percent change from baseline (95% confidence interval) in lipids, lipoproteins and hs-CRP

		**LDL-C**	**TC**	**TG**	**HDL-C**	**non-HDL-C**	**Apo B**	**Apo A-I**	**hs-CRP**
Non-obese	EZ/Simva (n = 77)	−25.2	−13.9	−4.8	1.8	−19.9	−16.6	2.0	−7.1
	Doubling statin (n = 42)	−4.9	−4.1	−5.3	−0.2	−4.8	−5.6	−2.3	−3.7
	Rosuva 10 (n = 42)	−17.4	−9.4	−2.0	2.6	−13.9	−11.6	1.3	−10.1
Obese	EZ/Simva (n = 237)	−21.6	−12.7	−6.1	1.2	−17.3	−13.7	−0.4	−1.5
	Doubling statin (n = 117)	−10.7	−5.3	−0.8	1.8	−8.2	−8.0	0.0	1.1
	Rosuva 10 (n = 249)	−20.7	−11.4	−4.2	1.6	−16.1	−12.3	0.6	−9.7

The safety and tolerability profiles were generally similar between treatment groups (Table 3). In the group of obese subjects, 9.9%, 9.7% and 6.8% of subjects experienced ≥1 AE in the ezetimibe/simvastatin group, in the doubling the statin dose group, and the rosuvastatin group, respectively. Three subjects each from the ezetimibe/simvastatin and the doubling the statin dose group discontinued due to an AE, and 2 were due to drug-related AEs. One subject experienced consecutive elevations in AST >3 X ULN in the rosuvastatin treatment group. There were 4 (2.2%), 1 (1.1%) and 3 (1.6%) reports of gastrointestinal-related AEs in subjects taking ezetimibe/simvastatin group, in subjects who doubled their statin dose, and in the rosuvastatin group, respectively; and 2 reports (1.0%) of hepatitis-related AEs in rosuvastatin treated subjects. In the non-obese subjects, 10.7%, 5.8% and 11.5% of subjects experienced ≥1 AE in the ezetimibe/simvastatin group, in the doubling the statin dose group, and in the rosuvastatin group, respectively. Four subjects from the ezetimibe/simvastatin group and 1 subject from the rosuvastatin group discontinued due to an AE and 1 was a drug related AE in the ezetimibe/simvastatin group. There were no reports of elevations in liver enzymes in the group of non-obese subjects. There were 6 (4.3%) and 4 (3.1%) reports of gastrointestinal-related AEs in subjects taking ezetimibe/simvastatin and in subjects taking rosuvastatin, respectively; and 2 reports (1.5%) of hepatitis-related AEs in rosuvastatin-treated subjects. No clinically meaningful differences in change from baseline in blood pressure between the treatment groups were observed in any subgroup.

**Table 3:** Summary of safety data (APaT population)

	**Obese subjects**			**Non-obese subjects**		
	**EZ/Simva 10/20 mg (N = 181)**	**Doubling statin dose (N = 93)**	**Rosuvastatin 10 mg (N = 192)**	**EZ/Simva 10/20 mg (N = 140)**	**Doubling statin dose (N = 69)**	**Rosuvastatin 10 mg (N = 131)**
	**n (%)**	**n (%)**	**n (%)**	**n (%)**	**n (%)**	**n (%)**
≥1 AE	18 (9.9)	9 (9.7)	13 (6.8)	15 (10.7)	4 (5.8)	15 (11.5)
Drug-related AE	6 (3.3)	2 (2.2)	2 (1.0)	2 (1.4)	0 (0.0)	0 (0.0)
Serious AE	0 (0.0)	1 (1.1)	2 (1.0)	2 (1.4)	0 (0.0)	0 (0.0)
Serious drug-related AE	0 (0.0)	0 (0.0)	0 (0.0)	0 (0.0)	0 (0.0)	0 (0.0)
Death	0 (0.0)	0 (0.0)	0 (0.0)	0 (0.0)	0 (0.0)	0 (0.0)
Discontinued due to						
AE	3 (1.7)	3 (3.2)	0 (0.0)	4 (2.9)	0 (0.0)	1 (0.8)
Drug-related AE	2 (1.1)	2 (2.2)	0 (0.0)	1 (0.7)	0 (0.0)	0 (0.0)
Serious AE	0 (0.0)	1 (1.1)	0 (0.0)	1 (0.7)	0 (0.0)	0 (0.0)
**AEs of special interest**						
Allergic reaction or rash	1 (0.6)	0 (0.0)	1 (0.5)	0 (0.0)	0 (0.0)	1 (0.8)
Gastrointestinal-related	4 (2.2)	1 (1.1)	3 (1.6)	6 (4.3)	0 (0.0)	4 (3.1)
Hepatitis-related	0 (0.0)	0 (0.0)	2 (1.0)	0 (0.0)	0 (0.0)	2 (1.5)
**Laboratory AEs of special interest**	**N = 177**	**N = 89**	**N = 188**	**N = 131**	**N = 69**	**N = 125**
ALT ≥3xULN, consecutive*	0 (0.0)	0 (0.0)	0 (0.0)	0 (0.0	0 (0.0)	0 (0.0)
AST ≥3xULN, consecutive*	0 (0.0)	0 (0.0)	1 (0.5)	0 (0.0	0 (0.0)	0 (0.0)
ALT and/or AST ≥3xULN, consecutive*	0 (0.0)	0 (0.0)	1 (0.5)	0 (0.0	0 (0.0)	0 (0.0)
CK ≥10xULN, single	0 (0.0)	0 (0.0)	0 (0.0)	0 (0.0	0 (0.0)	0 (0.0)

*Consecutive includes those patients with (a) measurement ≥3 x ULN observed at 2 or more consecutive visits (b) a single, last measurement ≥3 x ULN, or (c) a measurement ≥3 x ULN followed by a measurement < 3 x ULN that was taken more than 2 days after the last dose of study medication.

*AE* adverse event, *ALT* alanine aminotransferase, *AST* aspartate aminotransferase, *CK* creatine kinase, *EZ* ezetimibe, *Simva* Simvastatin, *ULN* upper limit of normal.

## Discussion

There have been relatively few studies that have assessed the comparative efficacy of the combination of ezetimibe/simvastatin with both atorvastatin and rosuvastatin in the same trial. This is the first report of the consistency of treatment effect of all three of these high-potency lipid-lowering therapies between subgroups of obese and non-obese diabetic patients in the same trial. With obesity reaching global epidemic proportions and its strong relationship to the development of CVD and atherogenic dyslipidemia, it is important to understand the potential utility of lipid lowering drugs in this population. In this *post hoc* analysis of subgroups of obese and non-obese diabetic subjects, treatment with ezetimibe/simvastatin 10/20 mg resulted in numerically greater reductions in LDL-C compared with rosuvastatin 10 mg only in the non-obese subjects, while the combination of ezetimibe/simvastatin 10/20 mg resulted in greater changes in LDL-C levels compared with doubling the statin dose in subjects in both subgroups regardless of baseline obesity status (obese/non-obese). In addition, a higher proportion of subjects attained all 3 specified treatment targets (LDL-C, non-HDL-C and Apo-B) with ezetimibe/simvastatin 10/20 mg treatment vs doubling the statin dose to atorvastatin 20 mg or simvastatin 40 mg and vs rosuvastatin 10 mg in both subgroups of obese and non-obese subjects. The overall safety and tolerability profile appeared generally comparable and consistent across subgroups and all treatment groups.

The dyslipidemia profile typically observed in obese and diabetic individuals is generally similar and includes high triglycerides (≥200 mg/dL), non-HDL-C, and Apo B levels, increased levels of small, dense LDL-C particles, although often, relatively normal levels of LDL-C, and low HDL-C levels (<40 mg/dL in men and <50 mg/dL in women) [7,8]. As expected, subjects in the obese subgroup had higher mean baseline triglycerides than subjects in the non-obese subgroup; however, these mean levels were lower than the 200 mg/dL level specified by the NCEP ATP III guidelines that would define patients as having mildly or moderately elevated triglycerides [4]. In addition, they did not have low mean HDL-C levels as would be expected based on typical dyslipidemia profiles in obese patients [7,8]. Finally, mean LDL-C levels in the obese subjects were comparable to those of the non-obese subjects, with mean levels already at or below 100 mg/dL in all treatment groups, but not reaching the target level of <70 mg/dL as specified by the NCEP ATP III guidelines for very high risk individuals. It is important to note that these baseline numbers reflect treated baseline levels, likely resulting from pre-study treatment and/or the 6-week run-in period during which subjects were treated with a starting dose of simvastatin (20 mg) or atorvastatin (10 mg) and during which they agreed to maintain an approved cholesterol- and glucose-lowering diet. This pre-study treatment may be why the typical dyslipidemia profile was not observed.

The *post hoc* analysis results from the subgroup of obese subjects were generally consistent with those of the prespecified analysis results from the overall population with regard to percent change from baseline in LDL-C [14]. Specifically, in the overall population, treatment with the combination of ezetimibe/simvastatin resulted in significantly greater reductions in LDL-C and other lipids compared with doubling the baseline statin dose to atorvastatin 20 mg or simvastatin 40 mg, but not compared with rosuvastatin 10 mg. However, in the subgroup of non-obese subjects in the current *post hoc* analysis, greater reductions in LDL-C were observed in favor of ezetimibe/simvastatin 10/20 mg vs doubling the baseline statin dose to atorvastatin 20 mg or simvastatin 40 mg and vs rosuvastatin 10 mg. These results are consistent with the sensitivity analyses of the overall population which showed statistically significant differences between the combination treatment and rosuvastatin 10 mg (−27.58 vs −22.20; *p* = 0.002). Moreover, the current sensitivity analysis results are consistent not only with the current exploratory analysis, but also with the primary analysis; however, for the ezetimibe/simvastatin 10/20 mg vs. rosuvastatin 10 mg comparison, the magnitude of the difference appeared to be somewhat higher compared with the main analysis. A previous *post hoc* analysis conducted in obese and non-obese subjects reported greater reductions with ezetimibe/simvastatin 10/20 mg vs. rosuvastatin 10 mg in both subgroups, however, those patients were not all diabetic [15]. In addition, the results of a study by Furman and colleagues in high risk patients (BMI = 30–31 kg/m^2^) who had not achieved LDL-C <100 mg/dL while treated with simvastatin demonstrated significantly greater reductions in LDL-C with the combination of ezetimibe/simvastatin vs rosuvastatin and vs atorvastatin (p <0.05) using average doses of 9/64 mg ezetimibe/simvastatin, 18 mg rosuvastatin, and 68 mg atorvastatin, which is consistent with the numerically greater reductions in most lipids vs doubling the statin dose to simvastatin 40 mg or atorvastatin 20 mg or vs rosuvastatin 10 mg observed in the current analysis [16]. One explanation for the inconsistencies between this group of subjects and those in previous studies may be differences in metabolism due to the presence of diabetes, which has been associated with high cholesterol synthesis and reduced cholesterol absorption efficiency regardless of obesity [17]. Larger trials to compare obese and non-obese diabetic patients are needed to fully assess these questions.

Although there is resounding evidence that LDL-C lowering reduces cardiovascular risk, there are also data to demonstrate that the typical dyslipidemia profile observed in diabetic patients, which is shared by obese patients, often results in residual risk even after LDL-C targets are achieved. Consequently, it is essential to consider secondary lipoprotein targets to reduce the atherogenic burden in diabetic patients once they have reached their individual LDL-C goal. Specifically, elevated Apo B and non-HDL-C are both recommended treatment targets for very high risk patients [4,9]. In this study, regardless of baseline obesity status (obese/non-obese), the combination of ezetimibe/simvastatin treatment resulted in higher percentages of diabetic patients achieving not only the aggressive LDL-C target of <70 mg/dL, but also non-HDL-C <100 mg/dL and Apo-B <80 mg/dL treatment targets compared with doubling the baseline statin dose and compared with rosuvastatin 10 mg. This result is consistent with a previous *post hoc* analysis of obese and non-obese subjects (of which only 1/3 were diabetic) in which higher percentages of patients achieved specified LDL-C, non-HDL-C and Apo B levels when treated with combination ezetimibe/simvastatin compared with rosuvastatin monotherapy [15].

The safety and tolerability profiles were generally consistent between treatments and between subgroups, although slightly more subjects taking ezetimibe/simvastatin 10/20 mg discontinued due to AEs compared with the other treatment groups. Previous trials comparing the safety profile of ezetimibe combined with simvastatin vs statins, including a *post hoc* analysis in obese and non-obese subjects do not suggest that there are significant tolerability differences between these treatments [15,18]; however, the use of the highest dose (80 mg) of simvastatin has been restricted by the US Food and Drug Administration due to the higher risk of myopathy/rhabdomyolysis [19]. Moreover, previous studies in high-risk diabetic subjects have not indicated tolerability issues with the combination treatment [10,11].

This study was an exploratory, *post hoc* analysis and did not include statistical comparisons, nor multiplicity adjustments. Moreover, the study was not powered to detect very rare adverse events and was of relatively short duration. Therefore, the efficacy and safety results should be interpreted with some caution.

These results suggest that regardless of baseline obesity status (obese/non-obese), switching to combination ezetimibe/simvastatin 10/20 mg provides a well-tolerated lipid-lowering effect in diabetic hypercholesterolemic subjects who have not achieved a goal of LDL-C <70 mg mg/dl (1.81 mmol/L) while on simvastatin 20 mg or atorvastatin 10 mg.

## Competing interests

MEH, RM, PB and JT are employees of Merck & Co, Inc. and may own stock or hold stock options in the company; JBR has a Research grant and has served on the Speaker's Bureau for Merck & Co., Inc.; JGJ received honoraria from Merck as a speaker for diabetes in Central America; PV received a research grant from Merck & Co, Inc., HV received a research grant from Merck & Co, Inc.

## Authors’ contributions

JR, JGJ, VP and HV collected or assembled the data, provided substantive suggestions for revision on subsequent iterations of the manuscript, and provided study materials or patients. RM conceived, designed or planned the study, performed or supervised analyses, interpreted the results, provided substantive suggestions for revision on subsequent iterations of the manuscript, and provided statistical expertise. MEH interpreted the results, wrote sections of the initial draft, and provided administrative, technical, or logistic support. PB conceived, designed and planned the study, and provided substantive suggestions for revision on subsequent iterations of the manuscript. JT interpreted the results, provided substantive suggestions for revision on subsequent iterations of the manuscript, and provided administrative, technical, or logistic support. All authors reviewed and approved the final version of the manuscript.
